# Palliative and end-of-life care on the battlefield: a scoping review

**DOI:** 10.1186/s13054-026-06359-1

**Published:** 2026-09-26

**Authors:** Chloé Matray, Olivier Duranteau, Anne Bercal, Paul Hebrard de Veyrinas, Mathieu David, Pierre Pasquier, Fabrice Gzil, Antoine Lamblin

**Affiliations:** 1 Department of Hepatology and Gastroenterology, Percy Military Teaching Hospital, Clamart, France; 2 Mobile Palliative Care Team, Percy Military Teaching Hospital, Clamart, France; 3 Department of Anesthesiology and Intensive Care, Percy Military Teaching Hospital, Clamart, France; 4 Department of Emergency and Intensive Care, Percy Military Teaching Hospital, Clamart, France; 5 French Military Medical Academy, École du Val-de-Grâce, Paris, France; 6 Allied Special Operations Forces Command SOFCOM, SHAPE, Casteau, Mons, Belgium; 7 Université Paris-Saclay, UVSQ, Inserm, CESP, Villejuif, 94807 France; 8 Espace éthique Île-de-France, Saint-Louis Hospital AP-HP, Paris, France; 9 Department of Anesthesiology and Intensive Care, Laveran Military Teaching Hospital, Marseille, France; 10 ⁠UMR 7268 ADÉS Aix-Marseille Université, EFS, CNRS, Marseille, France

**Keywords:** Palliative care, Military medicine, End-of-life care, Wounds and injuries, Pain management, Prolonged field care, Large-scale combat operation

## Abstract

**Background:**

Modern conflicts have brought about a paradigm shift in military medicine. In Prolonged Field Care, recommendations have so far focused on damage control and care aimed at ensuring survival. But, particularly in large scale combat operation, not all patients will survive, and despite the development of palliative care in the civilian sector, no extension of this approach has yet been envisaged for military medicine. This review aims to identify knowledge gaps in palliative and supportive care in combat situations.

**Methods:**

We conducted a literature review in PubMed, CAIRN, Scopus and Embase, with the last search carried out on 31 December 2025, using triangulation at each stage of the screening process. Studies published since 1900 focusing on frontline palliative and end-of-life care for combatants were included.

**Results:**

Of 3,629 unique records, 35 articles were included, ranging from World War II-era accounts to current prolonged field care guidelines. Pain management was the most extensively documented topic. Morphine, a historical mainstay, has been increasingly supplemented by oral transmucosal fentanyl citrate and ketamine. Ketamine was favored in austere settings for its hemodynamic stability and multi-route administration. Other analgesics, such as paracetamol and NSAIDs, were also briefly described. Regional anesthesia was recognized as an analgesic force multiplier, though never described in an explicitly palliative context. For expectant and dying casualties, a limited but consistent body of literature addressed opioid titration and palliative sedation, notably via continuous midazolam infusion. Diverse end-of-life-related symptoms were addressed, including nausea, dyspnea, secretions, and delirium. Non-pharmacological measures, such as thermal regulation, mouth care, disarming, and comfort items, were described as accessible across provider levels. Spiritual care and family communication, though addressed in few sources, emerged as important but under-formalized components. Overall, evidence specific to palliative and end-of-life battlefield care remains sparse and largely extrapolated from curative-care literature.

**Conclusion:**

Pain management in combat is a well-researched field, but one that is rarely examined in the specific context of end-of-life care. Similarly, there is a notable gap in the literature regarding palliative care in a combat context. Where such topics are addressed, they are often simply a transposition of civilian data. The lack of training and doctrinal guidance prevents the proper integration of this specialized care, which aims to ensure dignity for dying combatants and to reflect the values upon which military medicine is founded.

**Clinical trial number:**

The protocol was pre-registered on the Open Science Framework (OSF) on 8 December 2025 (DOI: 10.17605/OSF.IO/U9RSG).

**Supplementary information:**

The online version contains supplementary material available at https://doi.org/10.1186/s13054-026-06359-1.

## Background

Modern armed conflicts have fundamentally altered the landscape of battlefield trauma care. The scale of casualties in large-scale combat operations (LSCO) far exceeds that of recent counterinsurgency operations [1]. Over two years of combat, an estimated 140,000 soldiers were wounded and 70,000 killed on the Ukrainian side, representing a casualty burden roughly ten times greater than the total US losses across twenty years of the Global War on Terror [2, 3]. These realities undermine the ‘Golden Hour’ paradigm that has shaped military trauma medicine for two decades, leaving battlefield teams to manage casualties under prolonged evacuation delays and persistent resource constraints [1, 4].

Within NATO’s framework, Role 1 provides unit-level triage and pre-hospital care [5], capabilities potentially stretched well beyond their design limits [4]. While Prolonged Field Care (PFC) has provided a structured framework for managing casualties when evacuation is delayed, its guidance centers predominantly focused on hemorrhage control, airway management, and patient stabilization [6]. The care of patients for whom survival is no longer a realistic goal, constrained by catastrophic injuries or limited resources, has received little attention [7–9]. These casualties qualified as “expectant” require a form of support for which no clinical guidelines currently exist, leaving providers to navigate complex dilemmas spanning medical, ethical, and operational demands with no doctrinal framework to rely on [1, 10, 11].

The World Health Organization defines palliative care as an approach that improves quality of life by preventing and relieving suffering [12], a framework long established in civilian medicine. David et al. [13] refer to end-of-life care, Muir et al. [7] to compassionate care, and NATO doctrinal literature more recently to care of the dying [14] in a still unresolved semantic reflection. Despite growing recognition of this challenge, available evidence remains dispersed across emergency medicine and civilian palliative care literature, and to our knowledge no prior synthesis has addressed palliative and end-of-life practices specifically within this context.

We therefore conducted a scoping review to characterize the extent and nature of the available evidence on palliative care, symptom management, and end-of-life support for combat casualties whose injuries and operational resource constraints place them beyond the reach of immediate curative care, with the aim of identifying gaps to inform future research and doctrinal development.

## Methods

### Study design

We conducted a scoping review following the methodological framework refined by Levac et al. [15]. Results are reported in accordance with the Preferred Reporting Items for Systematic Reviews and Meta-Analyses Extension for Scoping Reviews (PRISMA-ScR) checklist [16] to ensure transparent and complete reporting of the search during the collection process. The protocol was pre-registered on the Open Science Framework (OSF) on 8 December 2025 (DOI: 10.17605/OSF.IO/U9RSG).

### Search strategy

A systematic search of PubMed, CAIRN, Scopus, and Embase was conducted independently by two reviewers (CM, OD). The full search strategies for all four databases are provided in Supplementary Table 1S.

### Study selection and data extraction

Both reviewers conducted their searches between 1 and 30 December 2025. Records identified through database searching were imported into Rayyan® [17] where duplicates were removed. Titles and abstracts were then screened according to the predefined eligibility criteria. Disagreements were resolved through discussion, with AL available for arbitration if consensus could not be reached.

Studies were included if they were (1) published from 1900 onwards and (2) focused on wounded, dying, or end-of-life military personnel, (3) addressing palliative care as such, or comfort care, pain management, (4) within the context of armed conflict. Full-text articles were excluded if not related to (1) palliative care, (2) pre hospital military combat context, (3) not written in English or French, (4) single-case reports without argumentation or description of treatment.

A standardized data extraction form was developed in Excel and piloted on five articles before full implementation. For each included study, extracted data included year of publication, country of the first author, study design, and outcomes. Thematic content variables were also charted, covering management of pain, anxiety, swelling, fever, nausea, dyspnea, secretions, sedation, spiritual care, family management. Data extraction was conducted manually by CM and AB with AL for triangulation. Given the wide range of heterogeneity among the included articles, no formal risk of bias assessment was performed.

## Results

### Study inclusion and characteristics

**Fig. 1:**
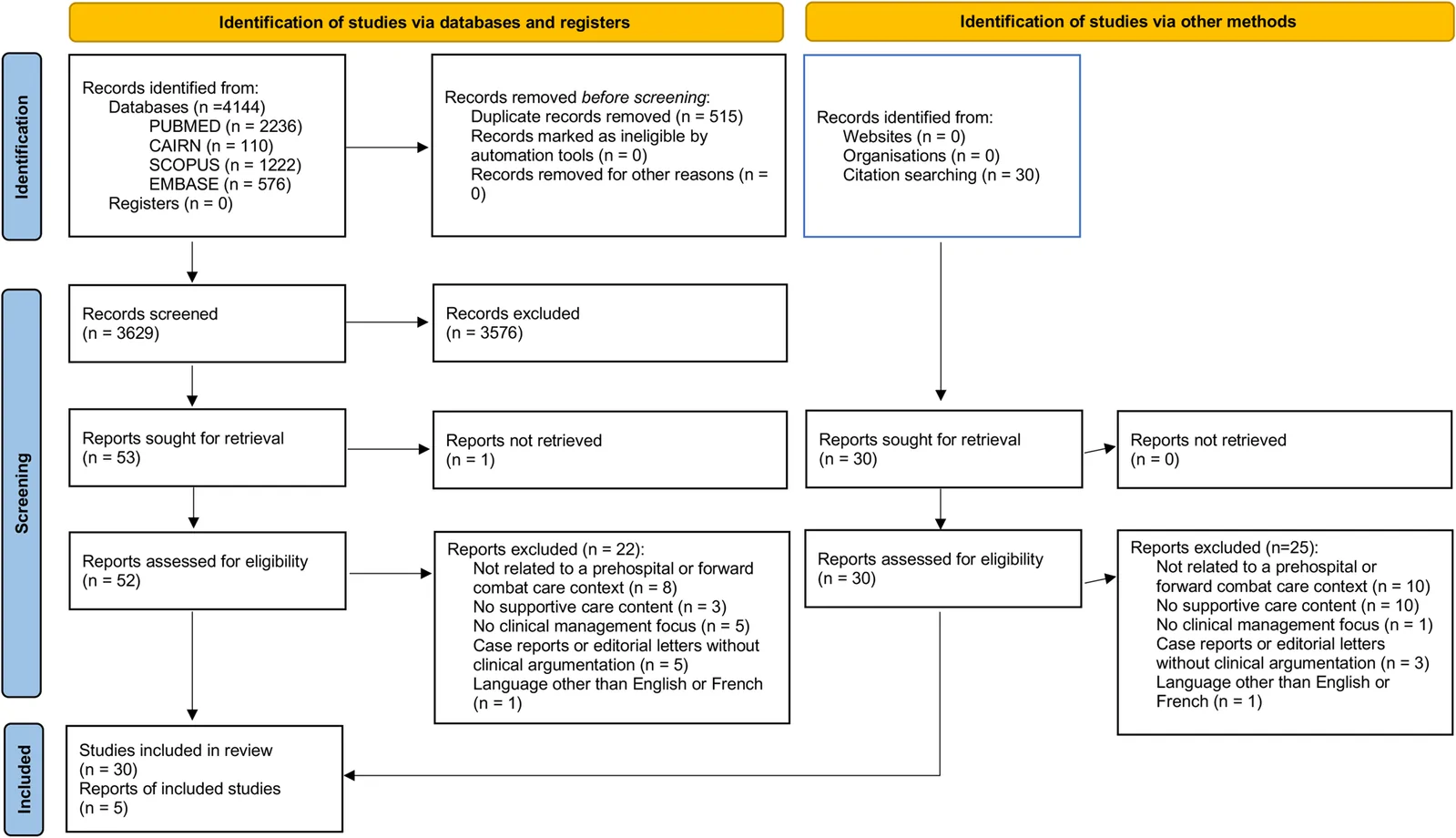
PRISMA flow diagram illustrating study selection for our scoping review

The database search retrieved 3,629 unique records, last completed on 31 December 2025. In total, 35 articles were included (Fig. 1). The full list of records in this scoping review can be found in Supplementary Table 2S.

### Pain management

#### Opioids

Morphine was the most extensively described agent, referenced from World War II [18] to contemporary guidelines [19–22]. Intramuscular (IM) morphine via autoinjector was the standard of care for decades [19, 23–25]. Its limitations were consistently documented with delayed onset in hemorrhagic shock, titration difficulties, and overdose risk with repeated dosing [23, 26–29]. Multiple guidelines subsequently recommended IV or alternative agents, supported by improvements in training and intraosseous (IO) devices [19, 22, 26–35].

Kotwal et al. [29] demonstrated in 22 Special Operations casualties that Oral transmucosal fentanyl citrate (OTFC) reduced pain scores from 9 to 3 within 15 minutes, with low adverse events, confirmed in a retrospective cohort of 286 casualties over seven years [35]. This effect was more readily achieved at night [29, 35]. OTFC was subsequently incorporated into Tactical Combat Casualty Care (TCCC) guidelines and issued to all UK frontline medical personnel as an adjunct to intramuscular morphine [19, 22, 23, 25, 30, 36]. Recommended administration was between the cheek and gum, repeated every 10 minutes, with the lozenge taped to the casualty’s finger or uniform as a safety measure if the consciousness were lost [19, 22, 28, 30, 34, 35, 37].

Intranasal fentanyl was not recommended in TCCC [30]. While intranasal butorphanol was documented as an alternative [37], a sufentanil sublingual tablet was proposed pending further refinement [30, 32, 34, 38].

For expectant and end-of-life casualties, PFC recommendations specify opioid administration until pain relief or, in non-communicative patients, until respiratory rate falls below 20 per minute, without hemodynamic restriction [7, 13, 21, 39]. The intramuscular route was not recommended [21]. David et al. expanded this with a structured table including morphine IV, IO or subcutaneous (SC) for 2–8 mg every 2–3 hours, hydromorphone (0.2–0.5 mg every 2–3 hours), and fentanyl (25–100 µg IV/IO every 30–60 minutes) [13].

#### Ketamine

Ketamine was extensively described across the corpus [19–22, 26–28, 30–32, 34, 37–44], incorporated into TCCC guidelines in 2012, with low-dose use recommended in the PFC setting [21]. Hudson et al. [45] found little difference between low-dose intramuscular ketamine and morphine in terms of performance impact. The emergence phenomenon, characterized by unpleasant dreams, was considered particularly relevant in noisy combat environments and managed effectively with small doses of midazolam [37, 43].

Intranasal ketamine was documented in a French retrospective case series of 140 casualties [42], and the 100 mg/mL concentration recommended, though field effectiveness was difficult to sustain without patient cooperation and a clear nasal cavity [30, 40]. Novel delivery systems proposed for future use included atomizer devices, an oral transmucosal lozenge, and intramuscular autoinjectors [19, 20, 30, 37, 44].

For expectant care, Reed et al. [39] proposed ketamine as a palliative agent, citing its familiarity to medics, its authorized use by all military providers and medics, and potential for sedation and anxiolysis alongside analgesia, while noting the absence of guidelines for continuous intravenous use in the PFC setting. David et al. [13] included ketamine for palliative analgesia at 0.1–0.2 mg/kg IV or 0.3–0.5 mg/kg IM every 30–60 minutes.

#### Other analgesics

Most of the included sources did not address non-opioid analgesics use specifically in an end-of-life context [19, 21, 22, 28–32, 34, 37, 40, 45]. Beecher recommended in 1946 aspirin and codeine as first-line agents for mild pain [18]. More recently, co-codamol or tramadol were described as oral analgesic options when other routes were not available [31, 45]. Parenteral or intranasal nonsteroidal anti-Inflammatory drugs (NSAIDs) extended the utility of this drug class where the oral route is unavailable [38, 44]. Intravenous paracetamol was documented as a possible adjunct in multimodal pain-management strategies, with opioid-sparing properties in the PFC setting [21, 38, 40]. Gabapentin was noted but with inconclusive evidence [28], and nitrous oxide-based gas for inhalation was mentioned for short painful procedures [31].

For end-of-life care specifically, acetaminophen 650 mg orally every 6 hours and ibuprofen 600–800 mg orally every 8 hours were described as components of symptom management [13].

#### Regional anesthesia

Regional anesthesia (RA) potential as an analgesic force multiplier was highlighted across several sources, freeing providers to attend concurrent casualties, though battlefield evidence was acknowledged as limited [18, 21, 26, 28, 31, 37, 38, 40, 41, 46, 47]. Laitselart et al. [47] advocated for its institutionalization in combat medicine, noting with Fisher et al. [26] that small volumes of local anesthetic could replace multiple opioid doses.

Historically, Beecher [18] documented intercostal, paravertebral, and local procaine blocks. UK protocols recommended lignocaine and bupivacaine for digital, femoral, and local infiltration blocks while advising against intercostal blocks on safety grounds [31].

Hematoma blocks combined with ketamine sedation were described as a useful field adjunct for fracture reduction [41]. More recently, fascia iliaca, intercostal, and suprascapular blocks performed before transport have been integrated into prehospital care [28], with contemporary PFC guidelines identifying peripheral nerve block as the preferred first-line analgesic for limb trauma, including supraclavicular, axillary brachial plexus, femoral, sciatic, and ankle blocks under ultrasound guidance [21, 37]. For continuous infusion, 0.2% ropivacaine was most commonly described, with clonidine as an occasional additive and patient-controlled analgesia as a feasible adjunct [38].

None of the included sources described RA in an explicitly palliative context.

#### Non-pharmacological analgesic interventions

Splinting and bandaging were documented as basic comfort measures accessible to all provider levels, with improvised splints constructed from available rigid materials where standard equipment was unavailable [18, 28, 37]. Burns were to be managed with dry, sterile dressings [27]. Cold therapy was mentioned in several forms, including compacted snow or cold water to cool injured extremities [28, 34, 37]. Battlefield auricular acupuncture (BFA) was also described as a rapid and low-risk technique, suitable for training [37, 38].

### Sedation

Sedation was predominantly documented in procedural and agitation management contexts. Beecher recommended barbiturates or paraldehyde for agitation and fear rather than morphine [18]. In curative settings, midazolam was preferred for residual agitation when pain was controlled [21, 30, 34], while ketamine was valued for its hemodynamic stability, preserved airway reflexes, and route flexibility [19, 20, 26, 34, 37, 39, 41, 43].

For expectant and end-of-life care, PFC guidelines stated that sedative administration represented a healthcare obligation even when vital sign depression was anticipated [21]. Muir et al. [7] identified sedation as a component of comfort measures for dying casualties. David et al. [13] provided a structured palliative sedation framework with midazolam continuous infusion at 1 mg/hour as the preferred agent. An alternative protocol was proposed, combining fentanyl 50 µg IV with ketamine 50 mg IV every 5 minutes until the desired level was achieved, either proportionate to alleviate an unpleasant symptom, or profound, to induce unconsciousness. Benzodiazepine options included midazolam, lorazepam, and diazepam, all titrated to clinical effect.

### Symptom management beyond pain and sedation

#### Nausea

Nausea management was documented in 8 sources [13, 19, 21, 22, 28, 30, 31]. Ondansetron was consistently recommended from 2014 onwards, with the oral route not advised operationally [19, 21, 22, 30]. Earlier UK protocols described cyclizine, metoclopramide, and prochlorperazine as interchangeable alternatives [31], and promethazine was noted for opioid-associated nausea [28]. A combined fentanyl-ondansetron lozenge was proposed for field use [19].

For end-of-life care, dexamethasone 4 mg IV/IO every 6–8 hours was recommended as a dual antiemetic and anti-inflammatory [13], and Muir et al. [7] proposed a palliative patch combining scopolamine with morphine to prevent nausea.

#### Airway secretions

Glycopyrrolate was documented for ketamine-induced hypersalivation in PFC guidelines [21], alongside atropine (0.5–1 mg IV/IO/IM every 6 hours) as antisialogogues for death rattle management in the only source to address secretion management in an explicitly palliative context [13].

#### Dyspnea

Dyspnea management was addressed in a single source [13], with documented regimens including morphine 2–8 mg IV/IO/SC every 2–3 hours or every 5–10 minutes as needed, hydromorphone 0.2–0.5 mg IV/IO/IM every 2–3 hours, and fentanyl 25–100 µg IV/IO/IM every 30–60 minutes. The oral route was not indicated given the clinical context.

#### Fever and swelling

Fever and edema management were documented across 4 sources [13, 18, 21, 34]. Acetaminophen and ibuprofen were identified as antipyretics, with ibuprofen and dexamethasone also referenced for edema [13, 21]. Beecher [18] highlighted lower extremity swelling in long-bone fractures as a preventable cause of prolonged pain, addressed by shoe removal and bandaging, while Fisher et al. [34] noted the historical use of ice and cold water for swelling reduction.

#### Anxiety and agitation

Joint Trauma System guidelines do not address anxiety management in the prolonged casualty care setting [39]. Clonidine was described as an anxiolytic and opioid-sparing agent in one source [28].

In a palliative context, benzodiazepines IV, IO or IM, including midazolam, lorazepam, and diazepam were recommended when anxiety or agitation persisted despite adequate analgesia, with doses titrated to clinical effect [13]. Basic verbal communication, including reassuring and explaining care, was identified as accessible to all providers [13, 21, 27], with allowing patients to express fears or end-of-life concerns further described as facilitating last wishes and acute psychological relief [13]. End-of-life delirium was addressed in a single source, which recommended haloperidol IV, IO or IM with 2,5-5 mg every 6 hours [13].

#### Other palliative specific measures

Thermal regulation and hypothermia prevention using blankets or specific equipment were proposed, alongside warmed intravenous fluids if required [19, 27]. Comfort measures for conscious dying casualties included water, food, and cigarettes [13, 21]. Noise reduction, light management, presence of familiar objects, and frequent reorientation were described as comfort interventions accessible to all provider levels [8, 13].

Beecher [18] identified mouth care as a basic but important comfort intervention in expectant and end-of-life care [8, 13].

Expectant casualties like any other severe casualty, or those under potentially disorienting medication required disarming as a safety precaution [19, 20, 27, 30].

### Spiritual and existential care

Spiritual and existential care was addressed in 3 sources. Grimell [48] described the professionalized role of Ukrainian chaplains providing pastoral care, sacraments, and end-of-life rites at stabilization points. Muir et al. and David et al. [7, 13] acknowledged spiritual needs as a palliative care component, noting that brief religious or cultural rituals helped with the process of loss, and that non-professional personnel could partially assume this role through basic acts of presence such as holding a hand or ensuring a dying soldier is not left alone [13].

### Family management

Communication with the family of a dying casualty was addressed in 3 sources [7, 8, 13]. McMillan [8] identified miscommunication with families as one of the most distressing elements. David et al. [13] recommended early, honest conversations regarding prognosis and care goals, transmission of last words via telecommunication or recorded media, respectful handling of remains, and psychological support. Muir et al. [7] similarly advocated for facilitated contact with next of kin whenever possible.

### Comprehensive supportive care: synthesis of current evidence

**Fig. 2:**
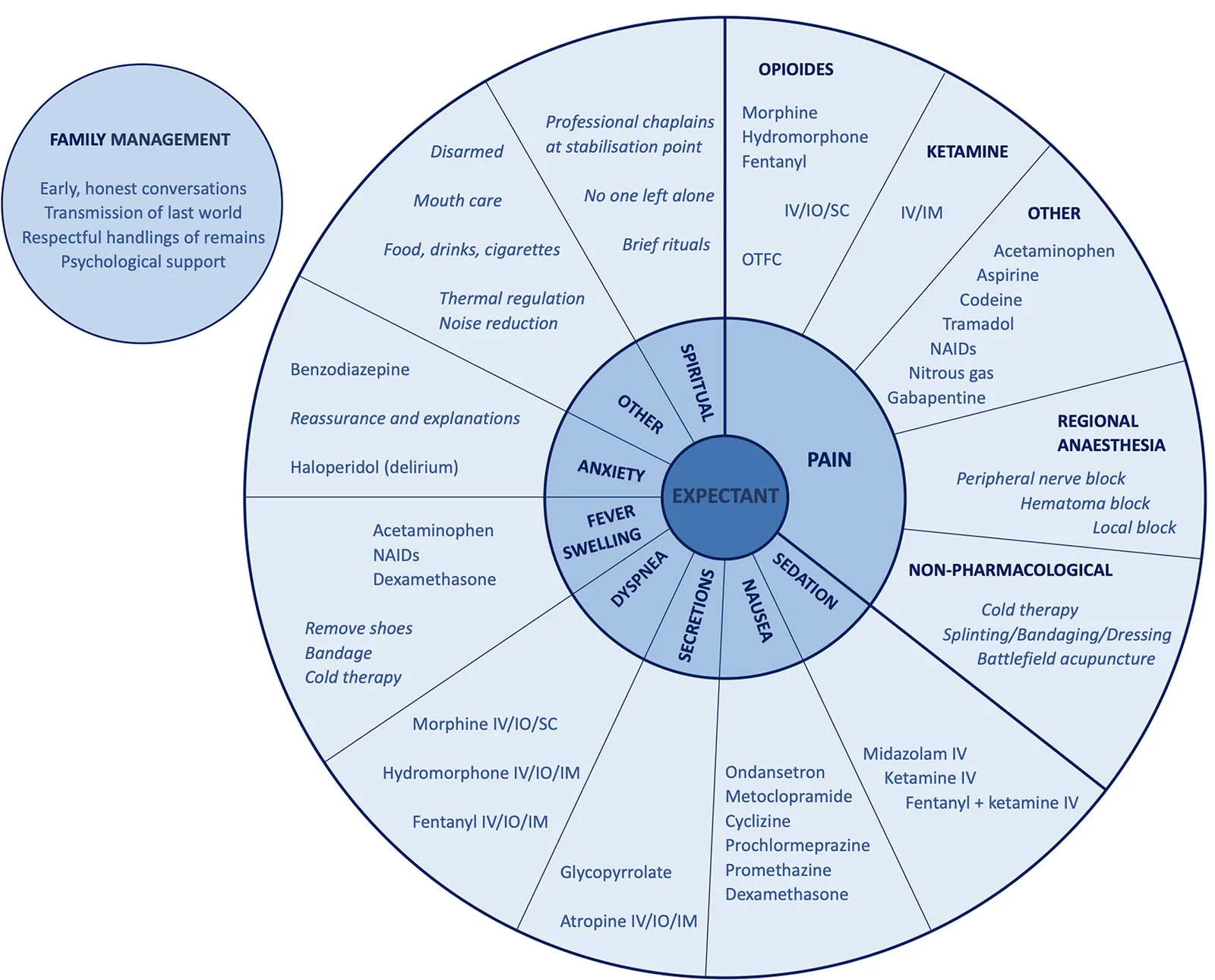
Mapping of the supportive care interventions identified in the literature for prehospital combat casualty care

The available literature supports a multimodal approach to supportive care for combat casualties beyond the reach of curative care, encompassing symptom control, communication, psychological and spiritual support, preservation of dignity, and family engagement when feasible (Fig. 2).

To operationalize these findings, Supplementary Table 3S summarizes possible palliative care medications and interventions according to care contexts and health care providers’ scope of practice.

## Discussion

The literature mapped in this review falls into two distinct bodies of work. The first, large and well-established, addresses pharmacological pain management in the context of combat casualty survival. The second, recent, sparse, and methodologically weak, addresses palliative and end-of-life care as an explicit clinical domain. The concentration of publications on pain management reflects the historical dominance of the curative paradigm and the Golden Hour doctrine [19, 21, 30]. While our search spanned 1900-present to capture this evolution, only three pre-2000 studies met inclusion criteria [18, 24, 31], with morphine as the predominant focus alongside emerging non-opioid and non-pharmacological approaches. Morphine has since been phased out of first-line TCCC analgesia for standard casualty care in favor of ketamine and non-opioid regimens [49], yet it remains the preferred opioid specifically within current PFC guidance for end-of-life care [21, 50]. Battlefield palliative care emerged as an explicit subject only in the most recent years, driven largely by the anticipated operational demands of LSCO [7, 8, 13].

The pharmacological management of combat pain is the best-documented area in this corpus. Morphine is progressively displaced in guidelines [19, 21, 22, 30]. Ketamine stands out as the most versatile and widely described agent, being almost described as an ideal combat analgesic [19, 28, 38]. Its trajectory from marginal use to guideline-endorsed first-line agent illustrates how doctrinal change can drive clinical practice at scale. OTFC, notable for its capacity for self- and buddy-administration directly relevant in LSCO has very recently seen its use severely restricted in the US [49]. Though still available in Europe, its withdrawal in the US led the TCCC Committee to reassess its recommendations in 2026, including the use of buprenorphine and esketamine for moderate to severe pain [49]. At last, regional anesthesia, though technically promising and logistically efficient, remains under-integrated into doctrine and training, representing a gap with both curative and palliative implications. These data partially address the pharmacological dimension of the research question. However, their use in a palliative rather than stabilization context, without hemodynamic endpoints, remains poorly documented and constitutes a major gap in the existing literature.

Several domains relevant to end-of-life care in combat settings were consistently absent or poorly represented across the included literature. Dyspnea, secretions, delirium, nausea, and fever are central symptoms in civilian palliative care therapeutic objectives [51–53]. David et al. [13] alone provides a structured pharmacological approach to these symptoms, extrapolated directly from civilian standards without adaptation to the operational context. The lack of consistent evidence regarding non-pharmacological care, particularly BFA, despite its use by US forces and others [54, 55], highlights an important gap and underscores the need for further research on basic comfort care in medication-depleted or austere environments. Psychosocial dimensions of care remain similarly underdeveloped. Although the included literature emphasizes the importance of facilitating communication with families [7, 8, 13], operational security considerations were not addressed. Notably, next-of-kin notification is typically a unit-level rather than a medical team responsibility, and may be impractical during active operations. Delayed transmission of written or recorded messages may therefore be preferable to live communication. Future guidelines should address secure and operationally feasible methods for family communication in combat settings, balancing ethical obligations with tactical constraints.

PFC guidelines’ statement that minimizing suffering is a healthcare obligation for expectant patients, remains the most explicit doctrinal affirmation of a palliative duty in the military literature [21]. Military medicine has not yet documented or integrated a comprehensive symptom-management approach into end-of-life care, a gap becoming operationally urgent [14]. The scale of these needs is also likely to vary with evacuation capability. Rapid evacuation may limit PFC to hours, reducing the duration and complexity of interventions required, whereas contexts similar to Ukraine may considerably prolonge PFC [56]. Operational feasibility further depends on the resources available to Medics. Ketamine and midazolam are commonly carried, whereas continuous infusion pumps for sedation are not standard equipment at Role 1 [30]. Non-pharmacological measures require few or no medical resources and should be feasible at the lowest levels of care, including for non-medical combatants. This may be particularly relevant for small, isolated teams such as Special Operations units, where the sole Medic-trained member may themselves become a casualty. While this scoping review does not aim to formulate clinical or operational recommendations, its findings support the need for a dedicated, tier-based integrated approach to end of life care, applicable across levels of care and deliverable by both warfighters and Medics, as a priority for future work.

The lack of a standardized definition of expectant casualties complicates their identification and classification [21, 57]. In Care Under Fire, survival may be temporarily unrealistic because of ongoing threats, whereas in PFC, expectant classification may instead result from resource constraints or delayed evacuation. Provider bias and moral distress may further influence these decisions, particularly under conditions of high operational stress [58]. Importantly, expectant status is not static and may evolve as tactical conditions, available resources, or evacuation capabilities change, underscoring the need for clearer, context-adaptive guidance to support healthcare providers in making these difficult decisions.

This guidance is currently absent from provider training. Most of the medical curricula contain no formal palliative care component and to our knowledge, no training program addresses specific scenarios where resuscitation fails and where care has to be delivered to a dying patient during hours, or days [8, 10]. Most exercises and simulations for LSCO training address technical competence rather than ethical end-of-life decision-making [59]. The cultural resistance to integrating death into military doctrine is probably the primary obstacle, more significant than logistical or pharmacological barriers. Battlefield palliative care is grounded in core military medical values, including the duty of care, non-abandonment, and preservation of dignity for all casualties regardless of prognosis [60]. These values may, at times, conflict with operational imperatives such as resource allocation to salvageable casualties, evacuation asset prioritization, and force protection. Resolving these tensions requires a framework that balances ethical obligations with operational realities. Current doctrinal frameworks should therefore evolve to better reflect this ethical complexity. In this regard, David et al. [13] proposed extending the principle of “leaving no one” beyond evacuation and survival to encompass dignity and relief of suffering at the end of life.

As military healthcare providers are at risk for moral distress related to unique ethical situations encountered [61], risk factors are associated with futility of care, bad death experiences, but also lack of resources and the need to ration care [61–63]. This gap places military providers at heightened risk of psychological injury. Lessons from civilian crisis care may offer transferable frameworks [64]. Addressing this through targeted psychological preparation, dedicated training and doctrine changes could significantly enhance battlefield resilience [49, 59].

Several concrete decision-support tools could facilitate this effort. Structured cognitive aids, such as pocket cards or quick-reference sheets, may promote rapid and standardized decision-making under operational stress [65]. A structured ethical framework such as the four-quadrant approach, already incorporated into UK military practice and NATO procedures, could be adapted for austere end-of-life scenarios [66, 67]. Integrating end-of-life scenarios into pre-deployment simulation and post-deployment debriefing may also facilitate emotional processing [68]. Finally, secure remote access to specialized military ethics or palliative care teams could provide timely expert guidance and help mitigate the psychological burden associated with these difficult decisions [21, 69].

### Limitations and strengths

To our knowledge, this scoping review is the first systematic synthesis of palliative practices in the prehospital combat setting. Several limitations should be acknowledged. The included sources are methodologically heterogeneous, with a near-absence of primary empirical studies. The corpus is dominated by US and UK military experience with limited representation of continental European perspectives, and of more recent conflicts including Ukraine, Israel, Lebanon, and Gaza. Most data derive from low-intensity conflicts whose relevance to the context of future LSCO remains uncertain. Potential exclusion of unpublished doctrine, and a palliative-specific corpus of only six sources further limit the conclusions that can be drawn.

### Implications for future research and doctrinal development

Several research priorities emerge from this review. Studies on the management of expectant casualties in simulated or real LSCO settings are needed, to develop palliative-specific protocols adapted to austere military environments. Finally, the integration of end-of-life care as a full domain within TCCC and PFC guidelines, rather than a marginal note on expectant patient management, should be pursued.

## Conclusion

Palliative care is well established in civilian settings, and every severely wounded combatant should be assured the best possible care. This review underscores the need to formally incorporate end-of-life care into military medical guidelines as a core component of casualty care, while integrating its principles into the training of both healthcare providers and combatants to ensure continuity of care across the battlefield. These priorities reflect a fundamental ethical imperative of military medicine: no combatant should die without dignity or relief from suffering. This review provides a foundation for future guidelines, training, and research aimed to achieve this standard.

## Take-home message

As future conflicts threaten to bring mass casualties on a scale that counterinsurgency warfare never approached, this scoping review reveals that current guidance on palliative and expectant battlefield care remains fragmented. By synthesizing the existing evidence and identifying key gaps, this review lays the foundation for standardized protocols to help providers deliver ethical, compassionate care to dying combatants.

## Electronic supplementary material

Below is the link to the electronic supplementary material.

Supplementary Material 1

Supplementary Material 2

Supplementary Material 3

## Data Availability

No datasets were generated or analysed during the current study.

## Abbreviations

BFA: Battlefield auricular acupuncture (BFA)
IM: Intramuscular
IO: Intraosseous
IV: Intravenous
LSCO: Large-scale combat operation
NSAID: Nonsteroidal Anti-Inflammatory Drugs
NATO: North Atlantic Treaty Organization
OSF: Open Science Framework
OTFC: Oral transmucosal fentanyl citrate
PRISMA-ScR: Preferred Reporting Items for Systematic Reviews and Meta-Analyses Extension for Scoping Reviews
PFC: Prolonged Field Care
RA: Regional anesthesia
SC: Subcutaneous
TCCC: Tactical Combat Casualty Care
