# Supplementary material for: Palliative and end-of-life care on the battlefield: a scoping review
Source: Crit Care. 2026 Sep 26;30:520. doi: 10.1186/s13054-026-06359-1 (PMC13644244; doi:10.1186/s13054-026-06359-1)
Supplement: Supplementary file 1 [file 13054_2026_6359_MOESM1_ESM.docx]

**PUBMED**

| BLOC 1 | CARE | 18/12/2025 | 30/12/2025 |
| --- | --- | --- | --- |
| #1 | palliative care [Majr] | 39052 |  |
| #2 | terminal care [Majr] | 47681 |  |
| #3 | patient comfort [Majr] | 370 |  |
| #4 | pain [Majr] | 310146 |  |
| #5 | withholding treatment [Mesh] | 16669 |  |
| #6 | palliative* [TIAB] | 94797 |  |
| #7 | comfort measure* [TIAB] | 618 |  |
| #8 | symptom* management [TIAB] OR symptom* relief [TIAB] | 28568 |  |
| #9 | pain management [TIAB] | 46316 |  |
| #10 | withdrawal of care [TIAB] | 287 |  |
| #11 | expectant* [TIAB] | 9574 |  |
| #12 | non-pharmacological [TIAB] | 18124 |  |
| #13 | non drug [TIAB] | 4332 |  |
| #14 | sedation [TIAB] | 53947 |  |
| #15 | compassionate care [TIAB] | 1716 |  |
| #16 | spiritual care [TIAB] | 3014 |  |
| #17 | end-of-life care* [TIAB] OR end-of-life measure* [TIAB] | 17301 |  |
| #18 | #1 OR #2 OR #3 OR #4 OR #5 OR #6 OR #7 OR #8 OR #9 OR #10 OR #11 OR #12 OR #13 OR #14 OR #15 OR #16 OR #17 | 587404 |  |
| BLOC 2a | CAREGIVER POPULATION |  |  |
| #19 | military medicine [Majr] | 23273 |  |
| #20 | military health [Majr] | 51 |  |
| #21 | military medical service* [TIAB] | 298 |  |
| #22 | military medic* [TIAB] OR military doctor [TIAB] OR military physician [TIAB] | 9490 |  |
| #23 | combat* [TIAB] OR combat medic* [TIAB] | 89629 |  |
| #24 | forward surgical team [TIAB] | 99 |  |
| #25 | non-medical personnel [TIAB] | 266 |  |
| #26 | buddy aid [TIAB] | 19 |  |
| #27 | combat lifesaver [TIAB] | 13 |  |
| #28 | emergency medical services [TIAB] | 12806 |  |
| #29 | #19 OR #20 OR #21 OR #22 OR #23 OR #24 OR #25 OR #25 OR #26 OR #27 OR #28 | 127626 |  |
| BLOC 2b | TREATED POPULATION |  |  |
| #30 | military personnel [Mesh] | 48081 |  |
| #31 | wounds and injuries [Mesh] | 1078800 |  |
| #32 | critical illness [Mesh] | 44412 |  |
| #33 | critically ill* [TIAB] | 71756 |  |
| #34 | soldier* [TIAB] | 14551 |  |
| #35 | combatant* [TIAB] | 8 |  |
| #36 | self-aid [TIAB] | 43 |  |
| #37 | dying patient [TIAB] | 1230 |  |
| #38 | #30 OR #31 OR #32 OR #33 OR #34 OR 35 OR #36 OR #37 | 1211451 |  |
| BLOC 3 | CONTEXT |  |  |
| #39 | warfare and armed conflicts [Mesh] | 50776 |  |
| #40 | military deployment [Mesh] | 128 |  |
| #41 | battlefield* [TIAB] | 3352 |  |
| #42 | tactical combat casualty care [TIAB] OR TCCC [TIAB] | 769 |  |
| #43 | prolonged casualty care [TIAB] OR PCC [TIAB] | 15883 |  |
| #44 | military* [TIAB] | 70961 |  |
| #45 | combat* [TIAB] | 89629 |  |
| #46 | deploy* [TIAB] | 97424 |  |
| #47 | war [TIAB] | 49462 |  |
| #48 | front-line [TIAB] | 10048 |  |
| #49 | austere environment* [TIAB] OR austere care [TIAB] OR austere military environment* [TIAB] | 852 |  |
| #50 | point-of-injury [TIAB] | 396 |  |
| #51 | pre-hospital [TIAB] | 7116 |  |
| #52 | prolonged field care [TIAB] | 169 |  |
| #53 | large scale combat operation [TIAB] OR LSCO [TIAB] | 123 |  |
| #54 | limited resource* [TIAB] OR resource-limited [TIAB] OR scarce medical resource* [TIAB] | 36421 |  |
| #55 | #39 40 OR #41 OR #42 OR #43 OR #44 OR #45 OR #46 OR #47 OR #48 OR #49 OR #50 OR #51 OR #52 OR #53 OR #54 | 377938 |  |
|  | FINAL COMBINAISON |  |  |
| #56 | #18 AND (#29 OR #39) AND #55 | 2233 | 2236 |

**CAIRN**

| ( | 19/12/2025 | 30/12/2025 |
| --- | --- | --- |
| soins palliatifs | 49991 |  |
| OU "fin de vie" | 8226 |  |
| OU "soins terminaux" | 710 |  |
| OU "soins de confort" | 405 |  |
| OU douleur | 35712 |  |
| OU sédation | 1788 |  |
| OU "arrêt des traitements" | 493 |  |
| OU "limitation des traitements" | 80 |  |
| OU "prise en charge des symptômes" | 127 |  |
| ) | 37366 |  |
| ET |  |  |
| ( |  |  |
| médecine militaire | 11763 |  |
| OU "personnel militaire" | 899 |  |
| ) | 1269 |  |
| ET |  |  |
| ( |  |  |
| guerre | 20838 |  |
| OU "conflit armé" | 11 |  |
| OU combat | 35049 |  |
| OU déploiement | 37107 |  |
| OU "champ de bataille" | 11360 |  |
| OU "milieu austère" | 8 |  |
| OU "ressources limitées" | 2981 |  |
| ) | 21435 |  |
|  |  |  |
| TOTAL | 111 | 111 |

**EMBASE**

| BLOC 1 | CARE | 22/12/2025 | 30/12/2025 |
| --- | --- | --- | --- |
| #1 | palliative care/exp | 166084 |  |
| #2 | terminal care/exp | 99239 |  |
| #3 | patient comfort/exp | 11824 |  |
| #6 | end-of-life care/exp | 99239 |  |
| #7 | palliative*:ti,ab. | 152565 |  |
| #8 | comfort measure*:ti,ab. | 21856 |  |
| #9 | (symptom* management OR symptom* relief):ti,ab. | 70158 |  |
| #10 | "pain management":ti,ab. | 64745 |  |
| #11 | "withdrawal of care":ti,ab. | 835 |  |
| #12 | expectant*:ti,ab. | 12034 |  |
| #13 | sedation:ti,ab. | 91657 |  |
| #14 | "compassionate care":ti,ab. | 2019 |  |
| #15 | "spiritual care":ti,ab. | 3518 |  |
| #16 | #1 OR #2 OR #3 OR #4 OR #5 OR #6 OR #7 OR #8 OR #9 OR #10 OR #11 OR #12 OR #13 OR #14 OR #15 | 541247 |  |
| BLOC 2a | CAREGIVER POPULATION |  |  |
| #17 | military medicine/exp | 31551 |  |
| #18 | military health/exp | 7251 |  |
| #19 | military medical service*:ti,ab. | 16632 |  |
| #20 | military medic*:ti,ab OR "military doctor":ti,ab OR "military physician":ti,ab. | 59799 |  |
| #21 | combat* [TIAB] OR combat medic*:ti,ab. | 20136 |  |
| #22 | "forward surgical team":ti,ab. | 105 |  |
| #23 | "non-medical personnel":ti,ab. | 397 |  |
| #24 | "buddy aid":ti,ab. | 25 |  |
| #25 | "combat lifesaver":ti,ab. | 14 |  |
| #26 | #17 OR #18 OR #19 OR #20 OR #21 OR #22 OR #23 OR #24 OR #25 | 108885 |  |
| BLOC 2b | TREATED POPULATION |  |  |
| #27 | military personnel/exp | 42391 |  |
| #28 | soldier*:ti,ab. | 16278 |  |
| #29 | combatant*:ti,ab. | 1046 |  |
| #30 | self-aid:ti,ab. | 59 |  |
| #31 | "dying patient":ti,ab. | 1654 |  |
| #32 | #27 OR #28 OR #29 OR #30 OR #31 | 52342 |  |
| BLOC 3 | CONTEXT |  |  |
| #33 | armed conflicts/exp | 38110 |  |
| #34 | military deployment/exp | 1968 |  |
| #35 | battlefield*:ti,ab. | 1023 |  |
| #36 | "tactical combat casualty care":ti,ab. OR TCCC:ti,ab. | 502 |  |
| #37 | "prolonged casualty care":ti,ab. OR PCC:ti,ab. | 21069 |  |
| #38 | military*:ti,ab. | 79829 |  |
| #39 | combat*:ti,ab. | 106505 |  |
| #40 | deploy*:ti,ab. | 123500 |  |
| #41 | war/exp | 38110 |  |
| #42 | front-line:ti,ab | 16898 |  |
| #43 | austere environment*:ti,ab. OR austere care:ti,ab. OR austere military environment*:ti,ab. | 822 |  |
| #44 | point-of-injury:ti,ab. | 495 |  |
| #45 | prolonged field care:ti,ab. | 197 |  |
| #46 | large scale combat operation:ti,ab. OR LSCO:ti,ab. | 99 |  |
| #47 | limited resource*:ti,ab. OR resource-limited:ti,ab. OR scarce medical resource*:ti,ab. | 67224 |  |
| #48 | #33 OR #34 OR #35 OR #36 OR #37 OR #38 OR #39 OR #40 OR #41 OR #42 OR #43 OR #44 OR #45 OR #46 OR #47 | 427102 |  |
|  | FINAL COMBINAISON |  |  |
| #49 | #16 AND (#26 OR #32) AND #48 | 1339 |  |
| #50 | #49 [embase]/lim | 576 | 576 |

**SCOPUS**

|  | 22/12/2025 | 30/12/2025 |
| --- | --- | --- |
| BLOC 1 : CARE |  |  |
| TITLE-ABS-KEY |  |  |
| palliativ* | 182222 |  |
| OR "terminal care" | 47775 |  |
| OR "patient comfort" | 15733 |  |
| OR "end of life care" | 21085 |  |
| OR "pain management" | 75449 |  |
| OR "comfort measure" | 754 |  |
| OR "symptom management" | 12414 |  |
| OR "symptom relief" | 10745 |  |
| OR "withholding treatment" | 12179 |  |
| OR "withdrawal of care" | 319 |  |
| OR expectant* | 12975 |  |
| OR nonpharmacologic* | 15250 |  |
| OR nondrug* | 2033 |  |
| OR sedation | 109222 |  |
| OR "compassionate care" | 2276 |  |
| OR "spiritual care" | 6859 |  |
|  | 460392 |  |
|  |  |  |
| BLOC 2 : POPULATION |  |  |
| TITLE-ABS-KEY |  |  |
| "military medicine" | 36044 |  |
| OR "military*" | 413037 |  |
| OR "combat medic*" | 517 |  |
| OR "forward surgical team" | 187 |  |
| OR "buddy aid" | 27 |  |
| OR "combat lifesaver" | 23 |  |
| OR soldier* | 76608 |  |
| OR combatant* | 7137 |  |
| OR "wounds and injuries" | 91262 |  |
| OR "critically ill*" | 100926 |  |
| OR "dying patient*" | 4538 |  |
| OR "self aid" | 84 |  |
| OR "non-medical personnel" | 389 |  |
|  | 641321 |  |
|  |  |  |
| BLOC 3 : CONTEXT |  |  |
| TITLE-ABS-KEY |  |  |
| war | 444443 |  |
| OR "military deployment" | 2681 |  |
| OR "armed conflict*" | 18157 |  |
| OR battlefield* | 21221 |  |
| OR combat* | 226571 |  |
| OR military* | 413037 |  |
| OR "front-line" | 20305 |  |
| OR "tactical combat casualty care" OR TCCC | 612 |  |
| OR "prolonged casualty care" OR PCC | 34190 |  |
| OR "prolonged field care" | 186 |  |
| OR "point of injury" | 497 |  |
| OR "austere care" | 8 |  |
| OR "austere military environment*" | 37 |  |
| OR "large scale combat operation" OR LSCO | 1497 |  |
|  | 1044389 |  |
|  |  |  |
| TOTAL | 1303 |  |
| English AND French | 1222 | 1222 |
