# Supplementary material for: Palliative and end-of-life care on the battlefield: a scoping review
Source: Crit Care. 2026 Sep 26;30:520. doi: 10.1186/s13054-026-06359-1 (PMC13644244; doi:10.1186/s13054-026-06359-1)
Supplement: Supplementary file 2 [file 13054_2026_6359_MOESM2_ESM.docx]

| **Title** | **Revue** | **Authors** | **Year of Publication** | **Country of the first author** | **Study Design** | **Outcome** |
| --- | --- | --- | --- | --- | --- | --- |
| A Novel Pain Management Strategy for Combat Casualty Care | Annals of Emergency Medicine | Russ S. Kotwal, Kevin C. O’Connor et al | 2004 | USA | Prospective observational | Evaluates oral transmucosal fentanyl citrate for acute pain reduction in wounded Special Operations soldiers in austere combat environment |
| A Triple Option Analgesia Plan for Tactical Combat Casualty Care | Journal of Special Operations Medicine | Frank K. Butler, Russ S. Kotwal et al | 2014 | USA | Clinical practice guidelines | Proposed Triple Option Analgesia: Combat Pill Pack, OTFC, Ketamine. |
| Acute Pain on and off the Battlefield: What We Do, What We Know, and Future Directions | International Anesthesiology Clinics | Michael L. Kent, Justin J. Upp et al. | 2011 | USA | Narrative review | Using this brief history of battlefield pain medicine as a framework, discussion of outcomes in battlefield pain management. |
| Anaesthesia in the Austere Environment | Journal of the Royal Army Medical Corps | AJ Mellor | 2005 | UK | Narrative review | Discusses anaesthesia in austere environments, deployed military. |
| Analgesia and Sedation Management During Prolonged Field Care | Journal of Special Operations Medicine | Jeremy Pamplin, Andrew D. Fisher, et al | 2017 | USA | Clinical practice guidelines | To described prolonged field care (PFC) role 1 guidelines. |
| Analgesia and Sedation for Tactical Combat Casualty Care - TCCC Proposed Change 21-02 | Journal of Special Operations Medicine | Andrew D. Fisher, Taylor T. DesRosiers et al | 2022 | USA | Clinical practice guidelines update with narrative review | Updated TCCC analgesia recommendations including sedation options. |
| Battlefield Analgesia - A Basic Approach | Journal of the Royal Army Medical Corps | G Hocking, WF de Mello | 1996 | UK | Protocol approach | To provide a basic approach to battlefield analgesia that can be used by the serviceman, either for self or buddy-buddy administration. |
| Battlefield Analgesia in Tactical Combat Casualty Care | Wilderness & Environmental Medicine | Ian S. Wedmore, Frank K. Butler Jr | 2017 | USA | Narrative review | Outlines evolution and evidence base for TCCC battlefield analgesia. |
| Battlefield analgesia: a brief review of current trends and concepts in the treatment of pain in US military casualties from the conflicts in Iraq and Afghanistan | Pain Management | Anthony Plunkett, Ali Turabi et al | 2012 | USA | Narrative review | To summarizes the more novel pain management approaches to combat casualties. |
| Battlefield Analgesia: An Advanced Approach | Journal of the Royal Army Medical Corps | G Hocking, WF De Mello | 1999 | UK | Expert consensus / guidelines | To provide a more advanced protocol, based on the drugs currently available operationally in Bosnia, for use on the battlefield or in disaster relief. |
| Battlefield pain management: A view of 17 years in Israel Defense Forces | Journal of Trauma and Acute Care Surgery | Avi Benov, Margaux M. Salas et al | 2017 | Israel | Retrospective observational registry cohort | To report the Israel Defense Forces Medical Corps experience with point of injury use of analgesia. |
| Care of the Dying Patient: Maximizing Compassionate Care on the Battlefield | Military Medicine | Kathryn B. Muir, Jeremy Edwards et al | 2025 | USA | Position statement / narrative review | To propose practical guidance for symptom management, communication, and compassionate care for dying battlefield casualties in LSCO. |
| Combat Casualties Treated With Intranasal Ketamine for Prehospital Analgesia : A Case Series | Journal of Special Operations Medicine | Christophe Dubecq, Romain Montagnon et al | 2023 | France | Retrospective case series | To report the prehospital use of IN ketamine for analgesia in penetrating trauma patients. |
| End-of-Life Care in the Austere Military Environment | Military Medicine | Jason David, Stevan Fairburn et al | 2025 | USA | Position statement / narrative review | To promote end-of-life care as doctrinally supported component of military medicine. |
| End-to-end military pain management | Journal of the Royal Army Medical Corps | D. J. Aldington, H. J. McQuay et al | 2011 | UK | Narrative review | Description of British Defence Medical Services pain management from POI to discharge |
| Is intramuscular morphine satisfying frontline medical personnels' requirement for battlefield analgesia in Helmand Province, Afghanistan? | British Journal of Pain | Tom N Blankenstein, Lorna M Gibson et al | 2015 | UK | Cross-sectional survey | To determine the opinions of frontline medical personnel on current intramuscular morphine use. |
| Ketamine for military prehospital analgesia and sedation in combat casualties | Journal of the Royal Army Medical Corps | Ross Moy, C Wright | 2018 | UK | Narrative review | To review the evidence, and outlines the future use of ketamine for provision of analgesia and seda- tion in combat casualties. |
| Ketamine Use for Palliative Care in the Austere Environment: Is Ketamine the Path Forward? | American Journal of Hospice & Palliative Medicine | John R. Reed, Stephanie K. Parks et al | 2025 | USA | Narrative review | To explore the use of ketamine in austere military environments for sedation and pain management of expectant casualties |
| Managing Pain on the Battlefield: Continuous Peripheral Nerve Blocks | Journal of the Royal Army Medical Corps | JG Hunter | 2010 | UK | Narrative review | Review of Continuous Peripheral Nerve Blocks evidence and feasibility in austere military environment. |
| Opioid analgesia on the battlefield: a retrospective review of data from Operation HERRICK | Journal of the Royal Army Medical Corps | Pip Lewis, C Wright et al | 2018 | UK | Retrospective cohort | To review the use of and complications  from opioid analgesics in a combat environment. |
| Pain in Men Wounded in Battle | Annals of Surgery | Henry K. Beecher | 1946 | USA | Cross-sectional observational | To review the treatment of pain in wounded men during the prolonged action on the Venafro and Cassino Fronts and later at the Anzio Beachhead and in France. |
| Pain Management in Current Combat Operations | Prehospital Emergency Care | Ian H. Black, John McManus | 2009 | USA | Narrative review | Overview of US military pain management in combat with challenges in austere environment |
| Pain Management in Military Trauma | Critical Care Nursing Clinics of North America | Kim Litwack | 2015 | USA | Narrative review | Overview of military trauma pain management from point of injury through rehabilitation. |
| Pain Management in the Wilderness and Operational Setting | Emergency Medicine Clinics of North America | Ian S. Wedmore, Troy Johnson et al | 2005 | USA | Narrative review with case examples | Discusses methods for treating pain in wilderness and operational deployed military settings. |
| Pain management in victims of conflict | Current Opinion Support Palliat Care | Dominic Aldington | 2012 | UK | Narrative review | To discuss developments in the prehospital environment, the field hospital, during repatriation and back in the home country. |
| Patterns of Palliation: A Review of Casualties That Received Pain Management Before Reaching Role 2 in Afghanistan | Military Medicine | Ian L. Hudson, Amanda M. Staudt | 2023 | USA | Retrospective cohort | To determine the proportion of casualties receiving analgesia over time. |
| Prehospital Analgesia and Sedation: A Perspective from the Battlefield | Current Trauma Reports | Andrew D. Fisher, Taylor T. DesRosiers et al | 2020 | USA | Narrative review | Provides battlefield perspective on prehospital analgesia and sedation |
| Prehospital Analgesia with Ketamine for Combat Wounds: A Case Series | Journal of Special Operations Medicine | Andrew D. Fisher, Bryan Rippee et al | 2014 | USA | Retrospective case series | To document the use of ketamineat the pount of injury in combat setting. |
| Prehospital Use of Ketamine in Battlefield Analgesia (Evidence Review) | Joint Trauma System / Committee on TCCC (Technical Report) | Nancy Dickey, Donald Jenkins, Frank K. Butler | 2012 | USA | Technical report / Narrative review | Defense Health Board's recommendations and review supporting ketamine addition to TCCC |
| Regional anaesthesia in combat settings: a position paper from military physicians | BMJ Military Health | Philippe Laitselart , S Hellander et al | 2025 | France | Expert consensus / position paper | To explore the benefits of RA in austere settings, the current barriers to its widespread adoption, and the required steps to institutionalise its practice. |
| Safety and efficacy of oral transmucosal fentanyl citrate for prehospital pain control on the battlefield | Journal of Trauma and Acute Care Surgery | Ian S. Wedmore, Russ S. Kotwal et al | 2012 | USA | Retrospective cohort | To analyzes the use of OTFC in the prehospital  battlefield environment by medical providers assigned to the US Army Special Operations Command. |
| Treating Pain on the Battlefield: A Warrior's Perspective | Current Pain and Headache Reports | Chester C. Buckenmaier III, Hisani Brandon-Edwards et al | 2010 | USA | Narrative review | Review of military pain management evolution. |
| Ukrainian Military Chaplaincy in War: An Introduction (Part 1) | Health and Social Care Chaplaincy (HSCC) | Jan Grimell | 2024 | Sweden | Qualitative interview study | To describe roles, tasks and development of Ukrainian military chaplaincy for spiritual care in combat and non-combat settings. |
| Use of ketamine for prehospital pain control on the battlefield: A systematic review | Journal of Trauma and Acute Care Surgery | Gaël de Rocquigny | 2020 | France | Systematic review | To analyze the content and quality of published data on the use of ketamine for prehospital pain management in military trauma. |
| Why we need to talk about deployed palliative care | BMJ Military Health | Kayleigh McMillan | 2021 | UK | Position statement / narrative review | To argues for inclusion of palliative care in deployed military medicine training and doctrine. |
