# Supplementary material for: Palliative and end-of-life care on the battlefield: a scoping review
Source: Crit Care. 2026 Sep 26;30:520. doi: 10.1186/s13054-026-06359-1 (PMC13644244; doi:10.1186/s13054-026-06359-1)
Supplement: Supplementary file 3 [file 13054_2026_6359_MOESM3_ESM.docx]

| Intervention | Drug  authorization | Providers | Care Under Fire | Tactical Field Care | Prolonged Field Care | Role 1 |
| --- | --- | --- | --- | --- | --- | --- |
| Morphine | + US/UK/FR | C, M, PM, Ph | - | + (UK/FR)  - (US 2026) | + | + |
| Oral Transmucosal Fentanyl (OTFC) | + UK/FR  - US (2026) | M, PM, Ph | - | - | + | + |
| Ketamine | + US/UK/FR | M*, PM*, Ph | - | + | + | + |
| Midazolam | + US/UK/FR | PM, Ph | - | + | + | + |
| Acetaminophene, NSAIDs | + US/UK/FR | C, M, PM, Ph | - | + | + | + |
| Ondansetron | + US/UK/FR | PM, Ph | - | + | + | + |
| Glycopyrrolate/Atropine | + US/UK/FR | PM*, Ph | - | - | + | + |
| Regional Anesthesia | + US/UK/FR | PM*, Ph | - | - | + (if trained/equipped) | + (if trained/equipped) |
| Non-pharmacological | N/A | C, M, PM, Ph | - | + | + | + |
| Spiritual care | N/A | Chaplains | - | - | - | - |
| Family communication | N/A | None | - | - | - | - |

**Table 3S : Possible palliative and symptom-management interventions by provider accessibility and Tactical Care Context.**

***Specifically trained advanced/SOF paramedic, according to national scope of practice / training**

**C : combatant ;** M : medic or trained combatant ; PM : paramedics ; Ph : physician ; IM : intramuscular ; IV : intravenous ; IO : intraosseous ; IN : intranasal ; PO : orally ; **NSAIDs** : Non-Steroidal Anti-Inflammatory Drugs
